# Effectiveness and Bioinformatics Analysis of Yiqi and Blood-Activating Therapy Combined with Chemotherapy in the Treatment of Non-Small Cell Lung Cancer

**DOI:** 10.3390/ph18101442

**Published:** 2025-09-25

**Authors:** Lu Xu, Weiling Lv, Ye Cheng, Chunjia Ping, Yizheng Wang, He Wang, Fan Lin

**Affiliations:** 1 College of Integrative Medicine, Fujian University of Traditional Chinese Medicine, Fuzhou 350122, Chinalvweiling66@163.com (W.L.); 18161231098@163.com (Y.C.); 17857315051@163.com (C.P.); 2007017@fjtcm.edu.cn (Y.W.); 2013057@fjtcm.edu.cn (H.W.); 2 Key Laboratory of Integrative Medicine on Chronic Diseases, Fujian Province University, Fuzhou 350122, China

**Keywords:** Yiqi and blood-activating therapy, non-small cell lung cancer (NSCLC), meta-analysis, data mining, network pharmacology, tumor immune microenvironment

## Abstract

**Objective:** To systematically evaluate the efficacy and explore the mechanisms of Yiqi and blood-activating traditional Chinese medicine (TCM) combined with chemotherapy in treating non-small cell lung cancer (NSCLC). **Methods:** Randomized controlled trials (RCTs) on chemotherapy combined with Yiqi and blood-activating TCM for NSCLC were retrieved from CNKI, VIP, Wanfang, and PubMed databases. The search period covered from the inception of each database to January 2025. Study quality was assessed via the Cochrane Risk of Bias tool. Meta-analysis evaluated clinical efficacy, data mining identified core herbs and active compounds, and network pharmacology analyzed targets and pathways. Immune infiltration analysis explored immunomodulatory mechanisms. **Results:** A total of 57 RCTs with 4865 patients were analyzed. Combined therapy significantly improved short-term efficacy, relieved symptoms (e.g., cough, fatigue), reduced adverse effects, and enhanced quality of life versus chemotherapy alone. Data mining identified *Astragalus membranaceus, Atractylodes macrocephala*, and *Poria cocos* as core herbs. Network pharmacology revealed 40 active compounds (including quercetin and kaempferol) that targeted 137 key proteins (e.g., TP53, AKT1), with these targets mainly involved in immune regulation. Immune infiltration analysis showed increased CD4+ T cells and balanced T cell subsets, indicating enhanced antitumor immunity. **Conclusions:** Yiqi and blood-activating TCM combined with chemotherapy improves NSCLC outcomes by modulating the tumor immune microenvironment. This supports the integration of TCM and highlights immune regulation as a key mechanism.

## 1. Introduction

Non-small cell lung cancer (NSCLC) accounts for approximately 85% of all lung cancer cases and remains one of the most prevalent malignancies worldwide. Lung cancer continues to lead in both incidence and mortality among cancers globally, posing a significant threat to public health. According to recent estimates, in 2022, there were approximately 2.5 million new lung cancer cases worldwide, representing 11.4% of all new cancer diagnoses. In the United States alone, the projected number of new lung cancer cases for 2025 is approximately 227,000, with an estimated 125,000 deaths. The predominance of NSCLC among these cases underscores its clinical importance and the urgent need for improved therapeutic strategies [1]. The high morbidity and mortality rates of NSCLC seriously threaten human health and life. Current treatment modalities for NSCLC include surgery, chemotherapy, targeted therapy, and immunotherapy. However, the overall prognosis for patients remains unsatisfactory, especially for those with intermediate and advanced-stage disease, where therapeutic effects are often limited and accompanied by significant adverse effects [2]. Therefore, exploring more effective and safer treatment strategies is crucial to improve the prognosis of NSCLC patients.

Traditional Chinese medicine (TCM) has shown distinct advantages in cancer treatment. Notably, the Yiqi and blood-activating method (benefiting Qi and activating blood) has been employed in the treatment of various tumors, demonstrating significant efficacy, particularly in alleviating clinical symptoms, improving quality of life, and reducing the adverse effects of radiotherapy and chemotherapy [3]. The Yiqi and blood-activating method aims to modulate the body’s internal milieu and bolster its intrinsic anti-tumor capacity by replenishing Qi, promoting blood circulation, and resolving blood stasis, thereby facilitating tumor treatment [4]. However, the precise role and underlying mechanisms of the Yiqi and blood-activating method in combination with chemotherapy for the treatment of NSCLC warrant further investigation.

Meta-analysis, a quantitative approach to reviewing existing literature, enhances statistical power and allows for more robust conclusions by synthesizing the results of multiple studies [5]. Data mining techniques facilitate extracting valuable information and identifying drug utilization patterns from extensive datasets. Complementarily, network pharmacology offers a comprehensive approach to analyzing the molecular mechanisms of TCM through the construction of a TCM-compound-target-disease network [6].

Based on the aforementioned context, this study aimed to systematically evaluate the clinical efficacy of the Yiqi and blood-activating method combined with chemotherapy in the treatment of NSCLC using meta-analysis. Subsequently, data mining was employed to identify the core herbs from the analyzed clinical herbal compounds. Finally, network pharmacology was utilized to investigate the mechanisms of action of these core herbs in the treatment of NSCLC.

## 2. Results

### 2.1. Meta-Analysis Results

#### 2.1.1. Screening of Literature

**Figure 1:**
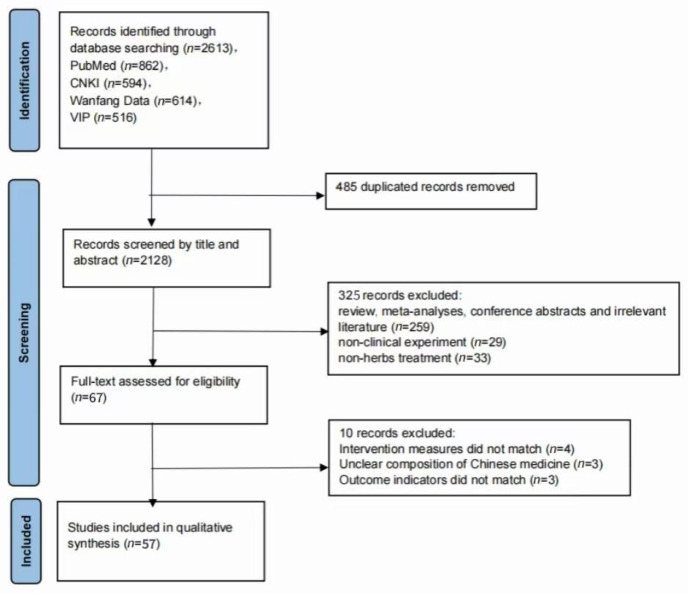
Flow diagram of study search.

A comprehensive search of four databases yielded a total of 2128 retrieved articles. After screening according to the inclusion and exclusion criteria, 57 articles were finally included in the meta-analysis. The literature screening process is shown in Figure 1.

A total of 4865 NSCLC patients were included in 57 randomized controlled trials (RCTs). The endpoints were categorized as follows: 43 endpoints evaluated recent efficacy, 23 endpoints described clinical symptomatic efficacy, 24 endpoints described Karnofsky Performance Status (KPS) scores, 23 endpoints analyzed leukopenia, 15 endpoints examined thrombocytopenia, 26 endpoints investigated gastrointestinal reactions, 10 endpoints reported CD3+ conditions, 12 endpoints described CD4+ conditions, 9 endpoints evaluated CD8+ cell status, and 13 endpoints assessed CD4+/CD8+ ratios. The basic profile of the final included RCTs is provided in Appendix A Table A1.

**Figure 2:**
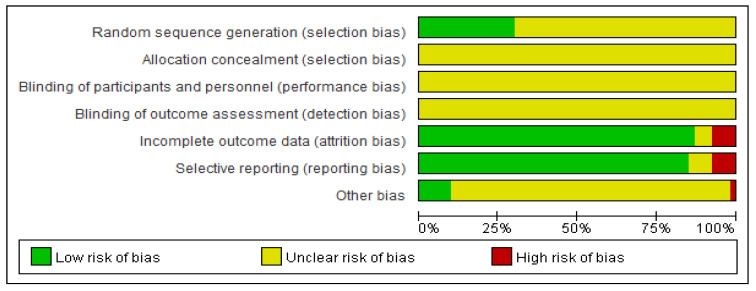
Risk of Bias Assessment.

Seventeen studies explicitly described using a “randomized number table” for participant allocation, but none reported allocation concealment or blinding. Among them, one paper documented a high patient dropout rate without specifying causes or management strategies, which was deemed to pose a high risk of follow-up bias. Additionally, some studies lacked detailed reporting of adverse reactions in their outcome measures, precluding accurate assessment of reporting bias risk. The specific risk-of-bias profiles for individual studies are shown in Figure 2.

#### 2.1.2. Meta-Analysis of Efficacy Outcome Indicators

As presented in Table 1, the treatment group demonstrated significant improvements over the control group across various efficacy outcome indicators. These included the recent effective rate (OR = 1.44, 95% CI = 1.33–1.66, *p* < 0.0001), clinical symptom efficacy (OR = 4.40, 95% CI = 3.48–5.55, *p* < 0.00001), incidence of adverse reactions, KPS scores, and changes in T-cell subpopulations. The short-term response rate (OR = 1.44, 95% CI = 1.33–1.66, *p* < 0.0001), clinical symptom efficacy (OR = 4.40, 95% CI = 3.48–5.55, *p* < 0.00001), and KPS score (WMD = 5.58, 95% CI = 4.69–6.47, *p* < 0.00001) of the treatment group were significantly better than those of the control group, and the incidence of adverse reactions was reduced.

Notably, extremely high statistical heterogeneity (I^2^ > 90%) was observed in the meta-analysis of T-cell subset changes, despite consistent effects in most therapeutic outcomes. This suggests significant inter-study variability, potentially arising from differences in patients’ baseline characteristics (e.g., age, disease duration), variations in Yiqi and blood-activating TCM formulas (composition, dosage), chemotherapy regimens (drug types, treatment courses), and methodological factors (sample size, outcome criteria). Such heterogeneity could limit the generalizability of the pooled effect estimates.

To address this, a random-effects model was employed to account for between-study variance. Sensitivity analyses (sequential exclusion of individual studies) and subgroup analyses (stratification by TCM formula or chemotherapy type) were performed where data permitted, yet residual heterogeneity remained. Therefore, interpretations regarding T-cell immunomodulation should be made with caution. Further high-quality, standardized studies—using uniform TCM formulas, clear chemotherapy protocols, and consistent outcome assessments—are warranted to clarify the sources of heterogeneity and validate the stability of therapeutic effects.

**Figure A1:**
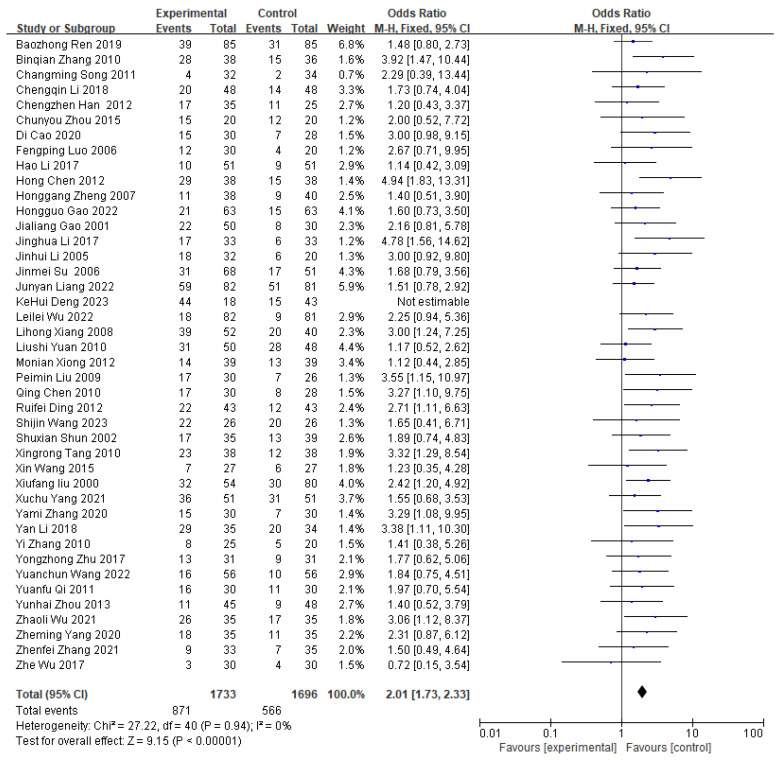
Forest plot: Impact of the Yiqi Huoxue method on short-term efficacy rate in the treatment of NSCLC. Forest Plot (right side graphic): The OR of each study is represented by a square (the size of the square corresponds to its weight), and the horizontal line represents the 95% CI; the diamond represents the pooled OR and its 95% CI.

**Figure A2:**
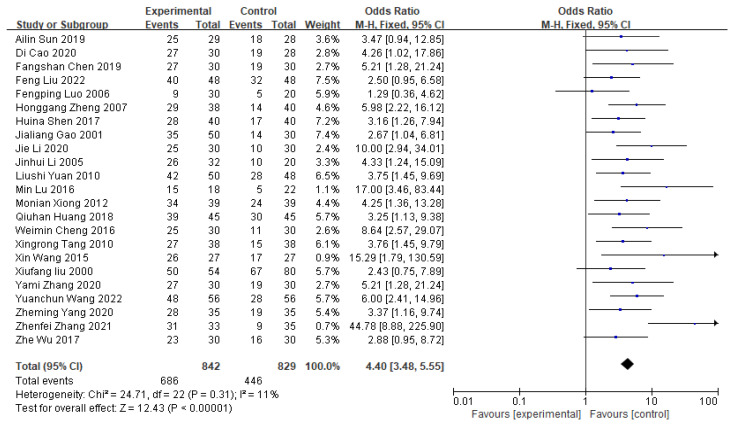
Forest plot: Effect of Yiqi Huoxue therapy on the efficacy of clinical symptoms in the treatment of NSCLC. Forest Plot (right side graphic): The OR of each study is represented by a square (the size of the square corresponds to its weight), and the horizontal line represents the 95% CI; the diamond represents the pooled OR and its 95% CI.

**Figure A3:**
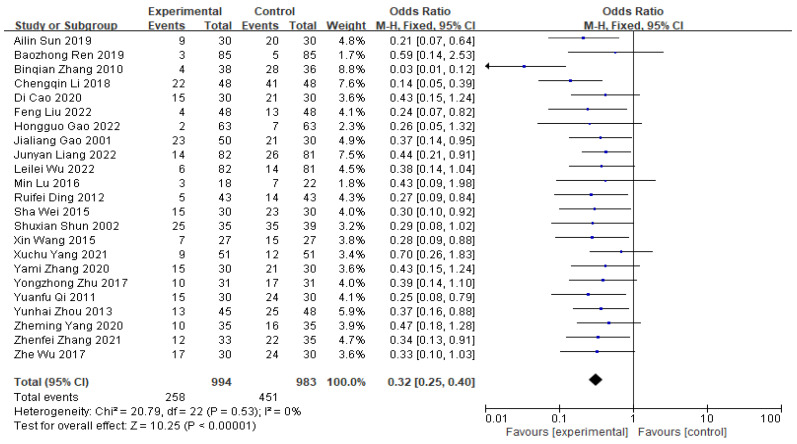
Forest plot: Effect of Yiqi Huoxue therapy on leukopenia in the treatment of NSCLC. Forest Plot (right side graphic): The OR of each study is represented by a square (the size of the square corresponds to its weight), and the horizontal line represents the 95% CI; the diamond represents the pooled OR and its 95% CI.

**Figure A4:**
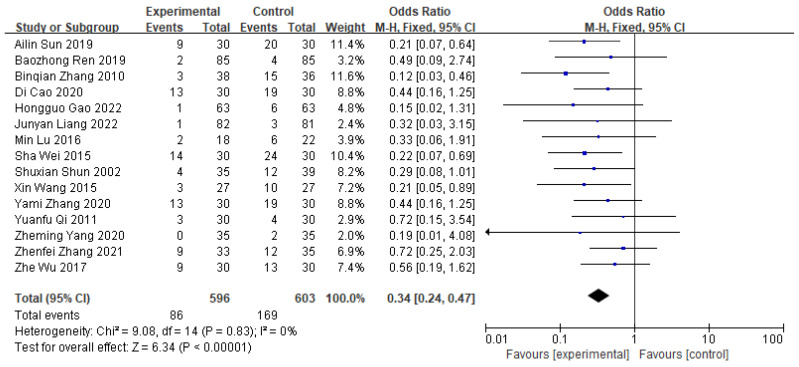
Forest plot: Effect of Yiqi Huoxue method on thrombocytopenia in the treatment of NSCLC. Forest Plot (right side graphic): The OR of each study is represented by a square (the size of the square corresponds to its weight), and the horizontal line represents the 95% CI; the diamond represents the pooled OR and its 95% CI.

**Figure A5:**
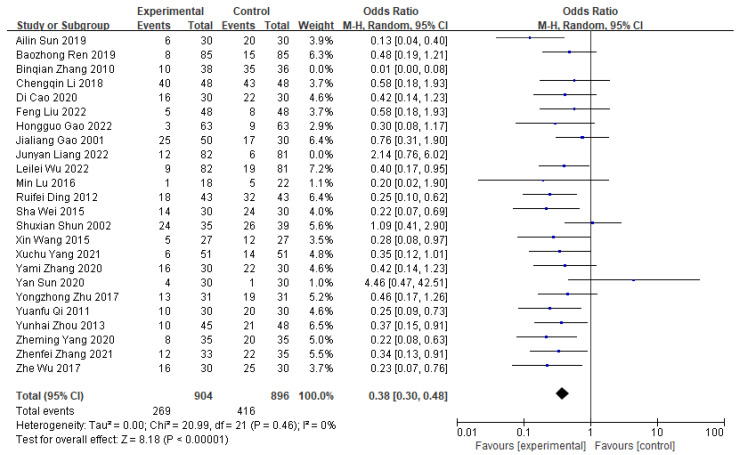
Forest plot: Effect of Yiqi Huoxue therapy on the incidence of gastrointestinal reactions in the treatment of NSCLC. Forest Plot (right side graphic): The OR of each study is represented by a square (the size of the square corresponds to its weight), and the horizontal line represents the 95% CI; the diamond represents the pooled OR and its 95% CI.

**Figure A6:**
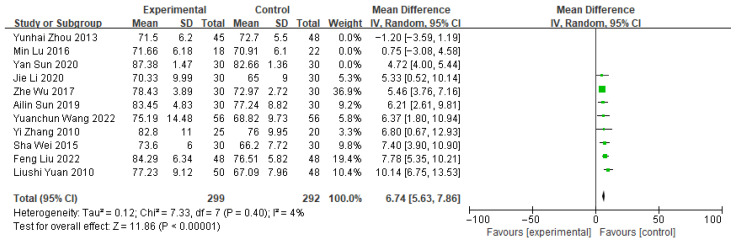
Forest plot: Effect of Yiqi Huoxue therapy on Karnofsky Performance Status (KPS) scores in patients with NSCLC. Forest Plot (right side graphic): The MD of each study is represented by a square (the size of the square corresponds to its weight), and the horizontal line represents the 95% CI; the diamond represents the pooled MD and its 95% CI.

**Figure A7:**
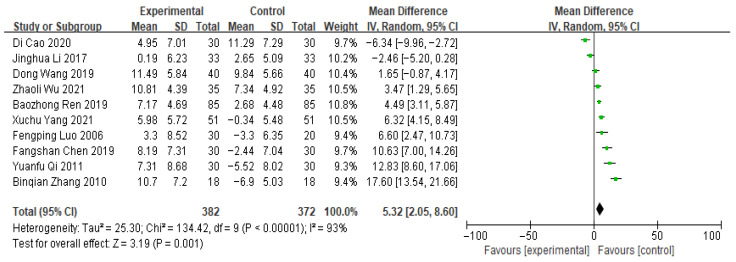
Forest plot: Effect of Yiqi Huoxue therapy on CD3+ levels in the treatment of NSCLC. Forest Plot (right side graphic): The MD of each study is represented by a square (the size of the square corresponds to its weight), and the horizontal line represents the 95% CI; the diamond represents the pooled MD and its 95% CI.

**Figure A8:**
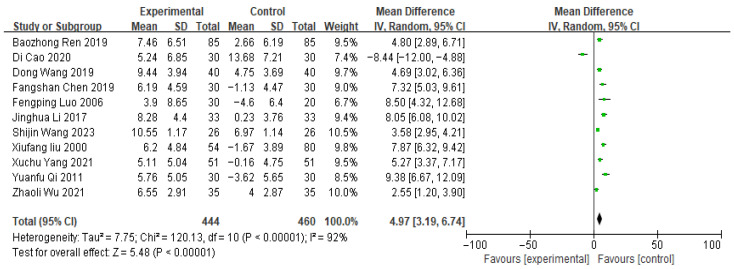
Forest plot: Effect of Yiqi Huoxue therapy on CD4+ levels in the treatment of NSCLC. Forest Plot (right side graphic): The MD of each study is represented by a square (the size of the square corresponds to its weight), and the horizontal line represents the 95% CI; the diamond represents the pooled MD and its 95% CI.

**Figure A9:**
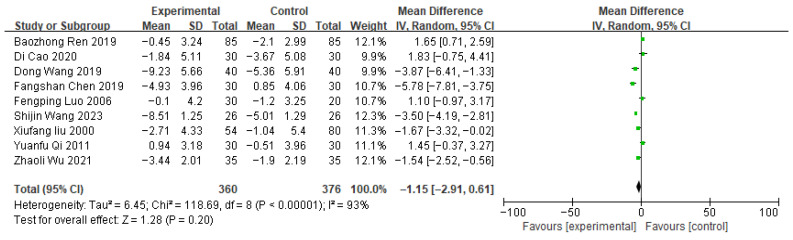
Forest plot: Effect of Yiqi Huoxue therapy on CD8+ levels in the treatment of NSCLC. Forest Plot (right side graphic): The MD of each study is represented by a square (the size of the square corresponds to its weight), and the horizontal line represents the 95% CI; the diamond represents the pooled MD and its 95% CI.

**Figure A10:**
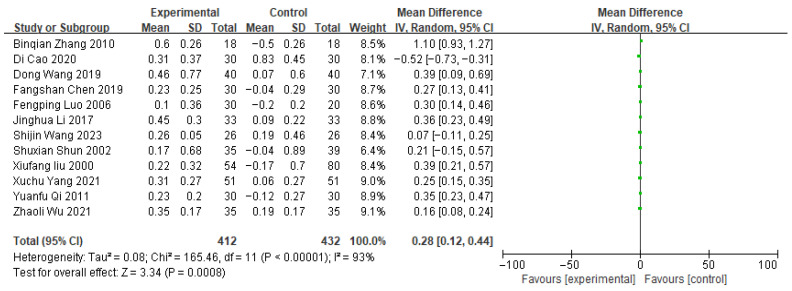
Forest plot: Effect of Yiqi Huoxue therapy on CD4+/CD8+ levels in the treatment of NSCLC. Forest Plot (right side graphic): The MD of each study is represented by a square (the size of the square corresponds to its weight), and the horizontal line represents the 95% CI.

In the treatment group, the elevations in immune markers CD3+, CD4+, and the CD4+/CD8+ ratio were significantly greater compared with those in the control group. Conversely, the level of CD8+ (WMD = −1.52, 95% CI = −1.94–−1.10, *p* < 0.0001) was lower than that in the control group. A detailed meta-analysis of the specific therapeutic outcome indicators is depicted in Figure A1, Figure A2, Figure A3, Figure A4, Figure A5, Figure A6, Figure A7, Figure A8, Figure A9 and Figure A10, which are included in the Appendix A.

### 2.2. Data Mining Results

#### 2.2.1. Drug Frequency Statistics

Drug frequency data were extracted from the 57 included studies: 149 single-herb medicines were used with a total frequency of 661. The frequency of each drug’s occurrence was sorted, and a total of 20 Chinese herbal medicines with a frequency of at least 10 appearances were identified as high-frequency drugs (i.e., the frequency of these herbs accounts for more than 20% of the total drug frequency). The top five herbs, in descending order of frequency, were *Astragali Radix* (Huangqi) (40 times), *Angelicae Sinensis Radix* (Danggui) (22 times), *Glycyrrhizae Radix et Rhizoma* (Gancao) (21 times), *Codonopsis Radix* (Dangshen) (20 times), and *Poria* (Fuling) (19 times). Subsequent high-frequency herbs included *Hedyotidis Diffusae Herba* (Baihuasheshecao) (18 times), *Atractylodis Macrocephalae Rhizoma* (Baizhu) (16 times), *Salvia miltiorrhiza* (Danshen) (16 times), *Curcumae Rhizoma* (Ezhu) (16 times), *Persicae Semen* (Taoren) (14 times), etc.

#### 2.2.2. Herb Association Analysis

**Figure 3:**
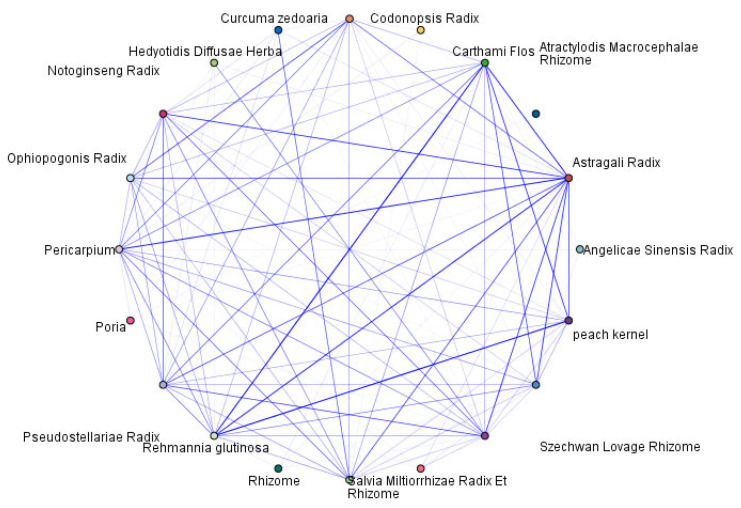
Correlation Network Analysis Diagram.

Association rule analysis of high-frequency drugs was conducted using the Apriori algorithm in SPSS Modeler 18.0 software. Parameters were set as minimum support ≥ 15%, confidence level ≥ 90%, and a maximum of two antecedents per rule. The association analysis results were ranked by rule support percentage, and the top five associations are listed in Table 2. The association network of high-frequency drugs is depicted in Figure 3.

#### 2.2.3. Cluster Analysis of Herb Use

**Figure 4:**
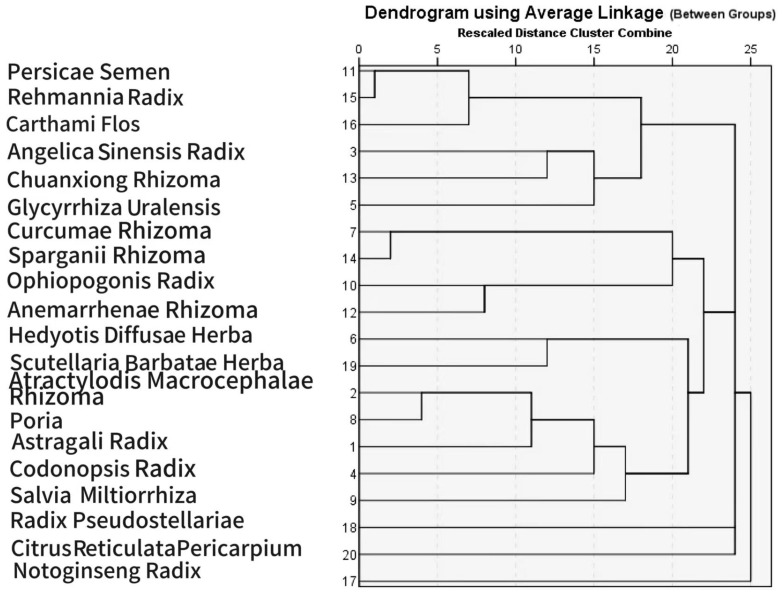
Cluster analysis of the most frequently prescribed herbs.

Hierarchical cluster analysis of the above high-frequency herbs was performed using SPSS Statistics 26.0 software. The intergroup linkage clustering method was employed, yielding six distinct clusters (Figure 4). The composition and characteristics of each cluster are as follows:

Class 1 (Blood-activating & stasis-resolving herbs): *Persicae Semen* (Taoren), *Rehmanniae Radix* (Shengdi), *Carthami Flos* (Honghua), *Angelicae Sinensis Radix* (Danggui), *Chuanxiong Rhizoma* (Chuanxiong).

Class 2 (Tumor-dispersing & qi-regulating herbs): *Curcumae Rhizoma* (Ezhu), *Sparganii Rhizoma* (Sanleng), *Ophiopogonis Radix* (Maimendong), *Anemarrhenae Rhizoma* (Zhimu).

Class 3 (Heat-clearing & detoxifying herbs): *Hedyotidis Diffusae Herba* (Baihuasheshecao), *Scutellariae Barbatae Herba* (Banzhilian).

Class 4 (Qi-tonifying & spleen-strengthening herbs): *Atractylodis Macrocephalae Rhizoma* (Baizhu), *Poria* (Fuling), *Astragali Radix* (Huangqi), *Codonopsis Radix* (Dangshen), *Salvia Miltiorrhiza* (Danshen).

Class 5 (Qi-regulating & damp-resolving herbs): *Radix Pseudostellariae* (Taizishen), *Citri Reticulatae Pericarpium* (Chenpi).

Class 6 (Blood-activating & hemostatic herb): *Notoginseng Radix* (Sanqi).

### 2.3. Network Pharmacological Results

#### 2.3.1. Identification of Action Targets for *Astragali Radix*, *Atractylodis Macrocephalae Rhizoma*, and *Poria* in NSCLC

**Figure A11:**
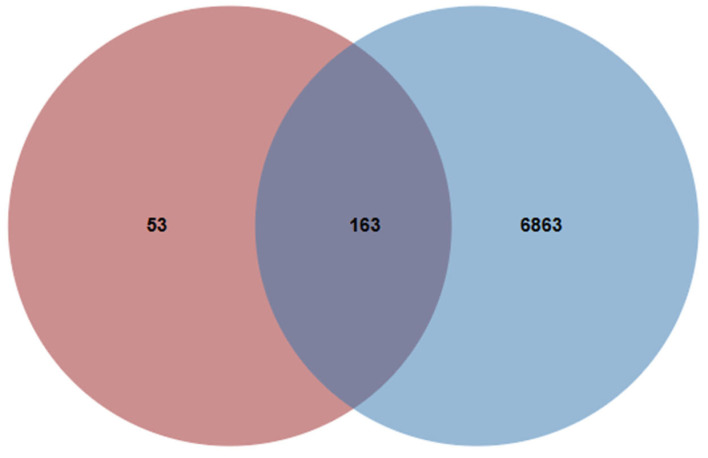
Drug Targets and NSCLC-Associated Proteins. The targets of the herbal combination *Astragalus*, *Atractylodes*, and *Poria* are depicted in red, and proteins related to NSCLC are highlighted in blue.

Following association and clustering analyses of the 20 high-frequency herbs, three key herbs-*Astragali Radix* (Huangqi), *Atractylodis Macrocephalae Rhizoma* (Baizhu), and *Poria* (Fuling)-were selected for further analysis. A total of 7207 NSCLC-related target proteins were retrieved from public databases (e.g., GeneCards, OMIM, and DRUGBANK databases). Active ingredients and their corresponding targets were then extracted from the Traditional Chinese Medicine Pharmaceutical (TCMP) database. *Astragali Radix* yielded 12 main active ingredients associated with 218 targets, *Atractylodis Macrocephalae Rhizoma* had 7 ingredients linked to 19 targets, and *Poria* contained 15 ingredients with 25 targets. After integrating and deduplicating the data, a final dataset of 40 unique active ingredients and 218 targets was established. By overlapping drug targets with NSCLC disease targets, 163 common targets were identified and used to create a Venn diagram (see Appendix A Figure A11).

#### 2.3.2. TCM-Compound-Target-Disease Network and PPI Analysis

**Figure 5:**
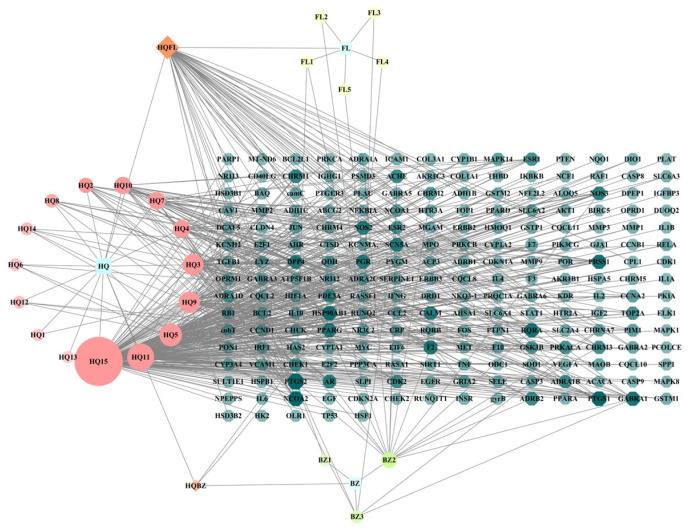
Network Analysis of Drug-Disease Targets in NSCLC. In the network diagram, dark green circular nodes represent potential targets, while blue nodes denote the herbs Scutellaria baicalensis (HQ), Poria cocos (FL), and Atractylodes macrocephala (BZ). The hexagonal nodes of various colors correspond to the active ingredients of these drugs.

The 40 active ingredients of *Astragali Radix*, *Atractylodis Macrocephalae Rhizoma*, and *Poria* and their 163 NSCLC-related targets were visualized in a Cytoscape network (Figure 5), consisting of 222 nodes and 541 edges. The top six highly connected compounds were quercetin, kaempferol, 7-O-methylisomucronulatol, formononetin, isorhamnetin, and 3-O-methylisomucronulatol, highlighting their critical roles in the Astragalus-Atractylodes-Poria combination for NSCLC therapy.

**Figure 6:**
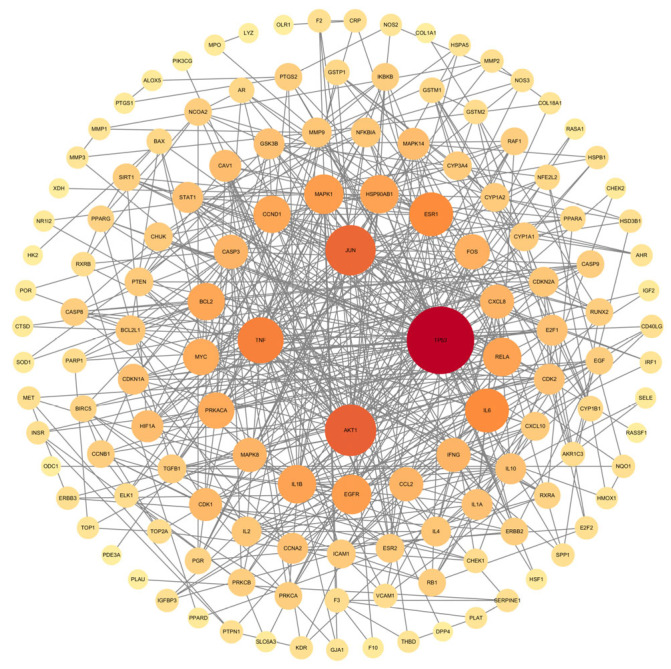
Core target PPI network diagram. Node size and color depth are proportional to the value of the degree.

The intersecting target information was uploaded to the STRING online platform to construct the compound-target protein–protein interaction (PPI) network for TCM in treating lung cancer. These data were then imported into Cytoscape for visualization, generating a core PPI network of targets composed of 137 nodes and 534 edges (Figure 6). Core target proteins were ranked by degree values, with the top 10 including TP53, AKT1, JUN, TNF, ESR1, IL6, MAPK1, EGFR, RELA, and HSP90AB1.

#### 2.3.3. Results of GO Functional and KEGG Pathway Enrichment Analysis

**Figure 7:**
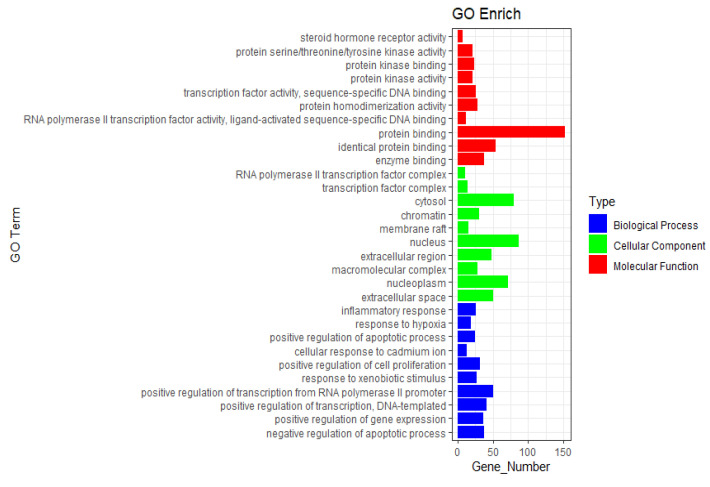
GO enrichment analysis.

The Gene Ontology (GO) functional analysis yielded 807 entries, categorized into biological processes (BP), cellular components (CC), and molecular functions (MF), with 86 and 149 entries for CC and MF, respectively. The top 10 entries from each category were selected to construct enrichment bar charts for CC, MF, and BP (Figure 7). The analysis indicated significant intersections in biological processes between *Astragali Radix*, *Atractylodis Macrocephalae Rhizoma*, *Poria*, and NSCLC. BP primarily included responses to inflammation and hypoxia. CC predominantly comprised the cytoplasm, nucleus, and extracellular space. MF mainly involved ligand-activated sequence-specific DNA binding, protein binding, and enzyme binding activities (Figure 7).

**Figure 8:**
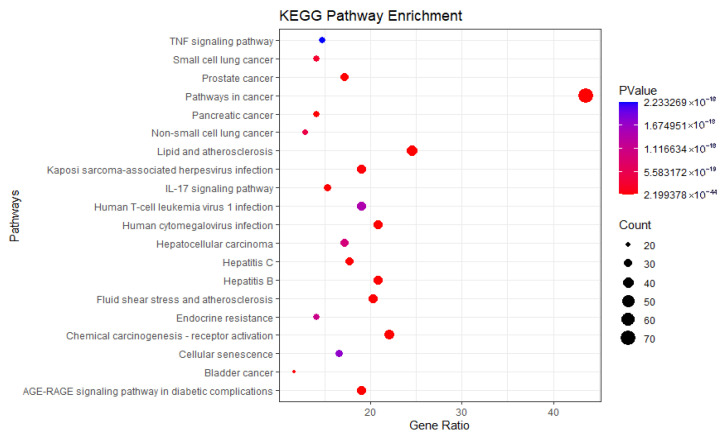
KEGG enrichment analysis.

Kyoto Encyclopedia of Genes and Genomes (KEGG) pathway analysis identified potential signaling pathways, including the TNF signaling pathway, IL-17 signaling pathway, and AGE-RAGE signaling pathway, among others (Figure 8).

### 2.4. Relationship Between Differential Gene Expression and Immune Infiltration

**Figure 9:**
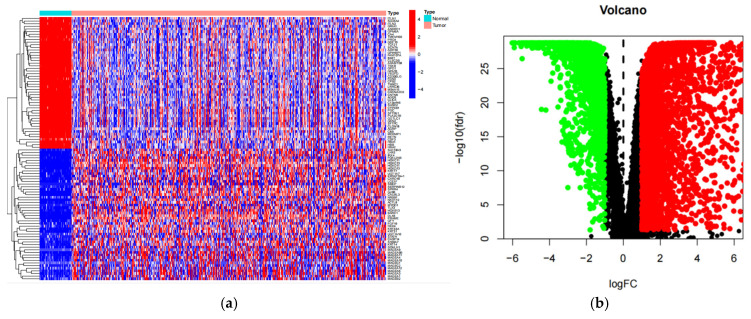
Heatmap (**a**) and Volcano Plot (**b**) of Differentially Expressed Genes. In the figure, black dots represent genes with no difference, green dots represent downregulated genes, and red dots represent upregulated genes.

In order to obtain a consensus of differentially expressed genes, gene expression quantification data and clinical information of lung adenocarcinoma (LUAD) patients in The Cancer Genome Atlas (TCGA) database were downloaded using TCGAbiolinks. Differentially expressed genes (DEGs) were screened using the “limma” package in R (version 4.3.3) with the criteria of |log2 fold change (FC)| > 1.0 and adjusted *p*-value < 0.05. Differentially expressed genes (DEGs) at the intersection of TCM-disease targets and LUAD were subjected to further analysis, yielding 69 DEGs with 39 upregulated and 30 downregulated genes (Figure 9). Expression profiling of core genes in NSCLC patients revealed significantly elevated expression of CCNA2, CDK1, and EGFR, whereas FOS, CCL2, and CAV1 exhibited reduced expression. Detailed expression data are presented in Table 3.

**Figure 10:**
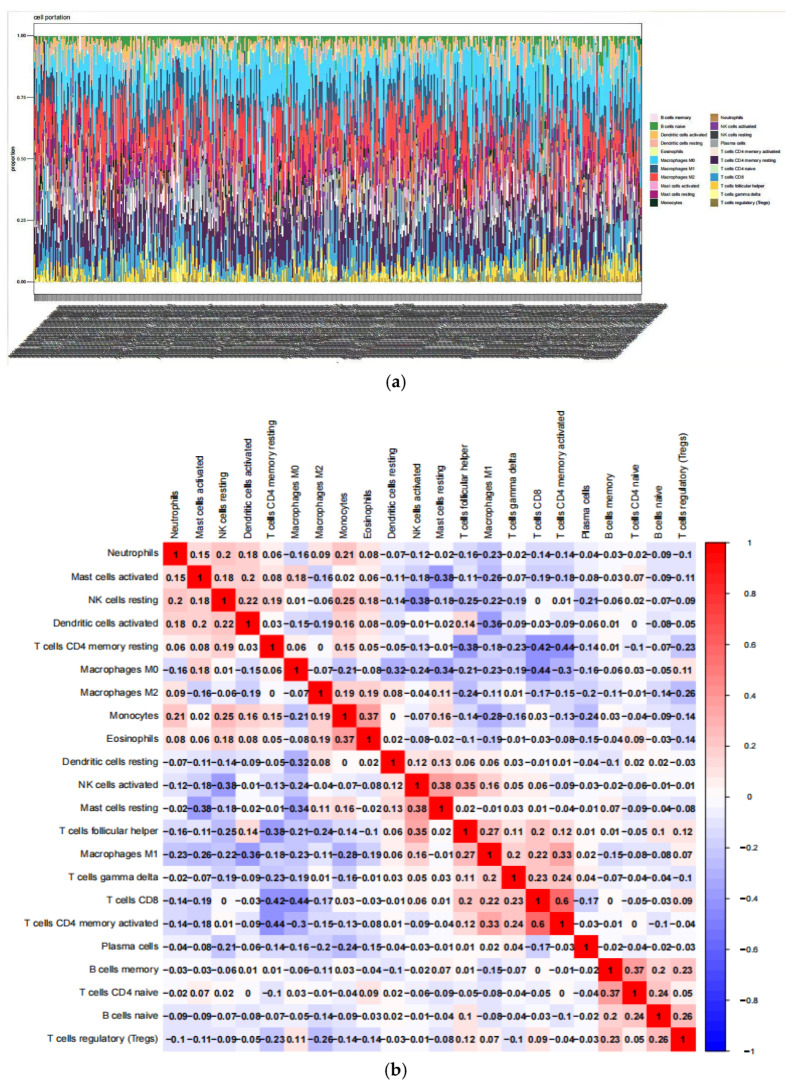
Correlation of Core Genes with Immune Infiltration Patterns. (**a**) Bar Chart of Immune Cell Proportions in Samples; (**b**) Correlation Matrix Plot of Immune Cells.

Subsequently, immune cell composition was analyzed using the CIBERSORT algorithm to estimate the proportions of 22 distinct immune cell types in each sample. Results showed that activated memory CD4+ T cells exhibited the strongest positive correlation with CD8+ T cells, while CD8+ T cells and M0 macrophages displayed the strongest negative correlation (Figure 10).

**Figure 11:**
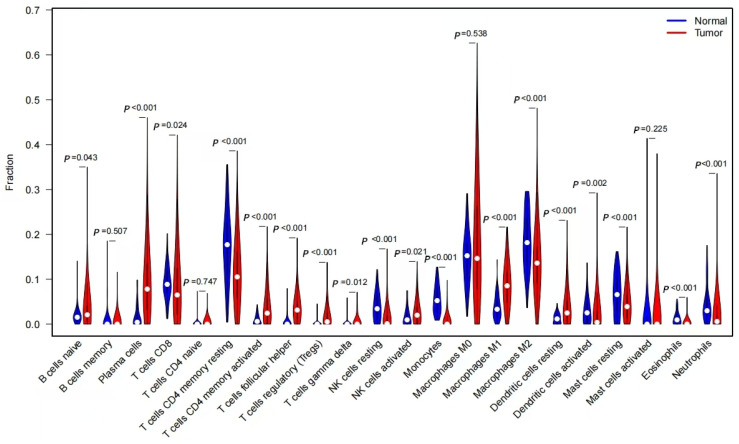
Analysis of the Relationship Between Core Genes and Immune Infiltration.

Boxplot analysis of immune cell distribution (Figure 11) revealed significant differences between healthy control and NSCLC patient samples (*p* < 0.05). Healthy controls had relatively higher abundances of resting memory CD4+ T cells (*p* = 0.002), resting natural killer (NK) cells (*p* = 0.013), and monocytes (*p* = 0.031), whereas NSCLC patients exhibited increased levels of plasma cells (*p* = 0.008), CD8+ T cells (*p* = 0.025), and activated memory CD4+ T cells (*p* = 0.004). These findings suggest that these immune cell populations may be significantly associated with NSCLC progression.

**Figure 12:**
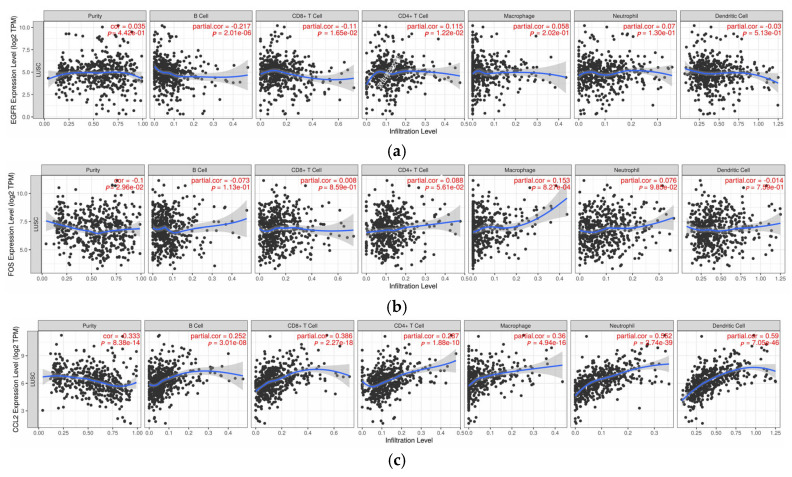

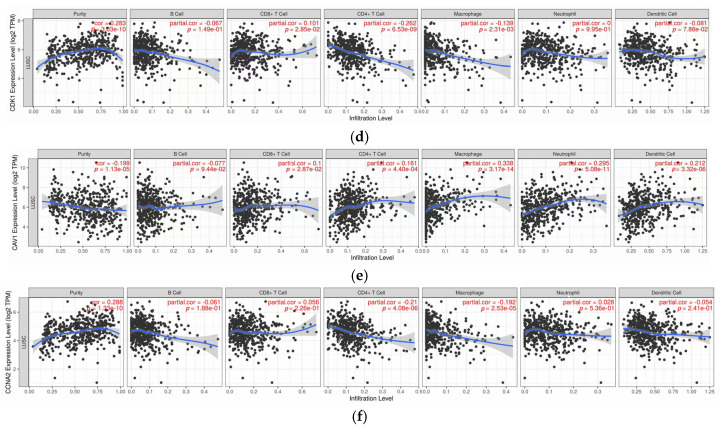
Scatter Plot Illustrating the Correlation Between Core Gene Expression and Immune Infiltrating Cells. (**a**) EGFR Expression Level; (**b**) FOS Expression Level; (**c**) CCL2 Expression Level; (**d**) CDK1 Expression Level; (**e**) CAV1 Expression Level; (**f**) CCNA2 Expression Level.

Using a significance threshold of *p* < 0.05, the expression levels of six key genes were positively correlated with CD8+ T cell infiltration, with correlation coefficients ranging from 0.088 to 0.272. The expression of EGFR (r = 0.24), FOS (r = 0.22), CCL2 (r = 0.18), and CAV1 (r = 0.15) also showed positive correlations with CD4+ T cell infiltration, with coefficients ranging from 0.082 to 0.24. Moreover, EGFR (r = 0.19) and FOS (r = 0.21) expression was positively associated with the infiltration of B lymphocytes, macrophages, neutrophils, and dendritic cells. Notably, granulocyte infiltration also exhibited positive correlations with these genes (Figure 12).

## 3. Discussion

In traditional Chinese medicine (TCM), non-small cell lung cancer (NSCLC) is primarily categorized as “lung fistula” (fei lou) or “lung accumulation” (fei bi). As elucidated in the Essentials of Medical Classics (Yixue Zhongzhong Canxi Lu), “Stasis formation arises from deficiency of zhengqi (vital qi), which allows pathogenic factors to invade.” This pathogenesis highlights a cascade wherein lung qi deficiency, impaired qi dispersion and descent, fluid metabolism disorders, phlegm accumulation, qi stagnation, and meridian obstruction progressively culminate in blood stasis. Over time, this vicious cycle exacerbates zhengqi deficiency, ultimately leading to toxin accumulation and tumorigenesis. Qi deficiency is thus central to NSCLC progression, with qi deficiency and blood stasis mutually reinforcing to drive disease development. While modern therapies such as surgery, chemotherapy, and targeted therapy effectively target tumor cells, they often concomitantly exacerbate qi deficiency and blood stasis [7]. Notably, clinical manifestations of qi deficiency and blood stasis are observed in over 60% of patients with advanced NSCLC [8]. Prolonged illness coupled with the adverse effects of antitumor treatments further deteriorates qi and blood stasis, impairing microcirculation and aggravating symptoms. Wang Qingren’s Corrections on Errors in Medical Works (Yilin Gaicuo) further states, “When vital qi is deficient, it fails to nourish the blood vessels, leading to stasis”—a concept congruent with modern observations that malignant tumor patients frequently exhibit immunocompromise, hypercoagulability, and peripheral microcirculation disorders [9]. Consequently, Yiqi and blood-activating therapy constitutes a critical therapeutic approach in NSCLC management, aiming to strengthen the healthy qi and resolve blood stasis.

While blood-activating herbs may exhibit dual effects on tumor progression—potentially promoting tumor growth when used as monotherapy—their integration within the Yiqi and blood-activating therapeutic framework may mitigate this risk and enhance antitumor efficacy [5]. Modern pharmacological studies have demonstrated that herbal formulas combining qi-promoting and blood-activating components can suppress tumor-associated immune tolerance and tumor-promoting inflammatory pathways, thereby inhibiting metastasis [10]. Emerging evidence suggests that combining qi-benefiting and blood-activating agents with chemotherapy not only suppresses tumor cell engraftment and proliferation but also enhances systemic immune function [11]. In the present meta-analysis of 57 RCTs, 10 studies reported CD3+ T cell profiles, 12 analyzed CD4+ T cells, 9 investigated CD8+ T cells, and 13 assessed CD4+/CD8+ ratios. To systematically evaluate the immunomodulatory effects of TCM in NSCLC, we selected T cell subsets (CD3+, CD4+, CD8+, CD4+/CD8+ ratio) as key endpoints. Results showed that the treatment group (Yiqi and blood-activating TCM combined with chemotherapy) exhibited significantly higher CD3+ and CD4+ T cell levels compared to the control group. Concomitantly, CD8+ T cell levels were significantly lower in the treatment group. This shift in T cell subset balance likely represents a key immunomodulatory effect of the combined therapy. Collectively, these findings suggest that Yiqi and blood-activating therapy enhances antitumor immunity by modulating T cell subset distribution, potentially overcoming chemotherapy-induced immunosuppression and improving overall treatment efficacy.

Importantly, a higher CD4+/CD8+ T cell ratio has been consistently associated in clinical studies with improved overall survival and better prognosis in NSCLC patients. This ratio reflects a more favorable immune milieu in which helper CD4+ T cells provide critical support for cytotoxic responses and immune memory. Therefore, the observed increase in the CD4+/CD8+ ratio in the treatment group not only indicates enhanced immune regulation but may also directly translate into improved patient outcomes. Such immunological shifts likely contribute to reduced tumor progression and prolonged survival, supporting the potential of Yiqi and blood-activating therapy as an adjunct to standard chemotherapy [12].

Data mining analysis identified the top 10 most frequently used herbs in NSCLC clinical practice as *Astragali Radix*, *Angelicae Sinensis Radix*, *Glycyrrhizae Radix et Rhizoma*, *Poria*, *Atractylodis Macrocephalae Rhizoma*, *Salviae Miltiorrhizae Radix*, *Curcumae Rhizoma*, and *Persicae Semen*. Association rule analysis revealed that *Astragali Radix*, *Atractylodis Macrocephalae*, *Poria*, and *Ginseng Radix* (7 herbs in total) were frequently combined to treat Spleen-Qi deficiency syndrome [13]. In this study, core drugs were selected based on frequency and correlation, with the Astragali Radix-Poria herb pair demonstrating the strongest association. Clinically, these herbs emphasize qi-benefiting, yin-nourishing, and spleen-strengthening effects through simultaneous tonification of the lung and spleen, alongside heat-clearing, detoxifying, and phlegm-resolving actions. *Astragali Radix* (most frequently used), sweet in taste and slightly warm in nature, acts on the lung and spleen meridians to replenish qi, elevate yang, stabilize the exterior, and arrest sweating [14].Modern pharmacology shows it promotes lung cancer cell apoptosis, inhibits proliferation/migration, modulates immunity, and reverses drug resistance [15]. *Atractylodis Macrocephalae Rhizoma* strengthens the spleen-stomach and eliminates dampness [16], while *Poria* tonifies the spleen, resolves dampness, promotes diuresis, and calms the mind [17].

The pathogenesis of NSCLC in TCM involves complex interactions between deficiency-excess and cold-heat patterns. When applying the theory of blood stasis, the selection of warming or cooling herbs adheres to the fundamental principle of “treating cold patterns with heat and heat patterns with cold”. This strategy integrates heat-clearing anticancer agents with spleen-lung tonics to counter drug resistance. For example, warm qi-tonifying herbs (*Astragali Radix*, *Atractylodis Macrocephalae Rhizoma*) are paired with cool heat-clearing herbs (*Scutellariae Barbatae Herba*, *Hedyotidis Diffusae Herba*) to balance pathogenic factors while strengthening the body [18]. This holistic approach addresses NSCLC’s dual nature of toxin accumulation and qi deficiency, providing a theoretical basis for personalized TCM-chemotherapy integration.

This study further revealed that TCM herbs such as *Astragali Radix*, *Atractylodis Macrocephalae Rhizoma*, and *Poria* contain diverse active compounds that may exert synergistic antitumor effects through multi-target mechanisms. For instance, quercetin and kaempferol in *Astragali Radix* play pivotal roles in regulating NSCLC-related targets. Quercetin inhibits proliferation and invasion of lung adenocarcinoma A549 cells by suppressing the STAT3 signaling pathway [19], while kaempferol—with established anticancer, antiviral, and anti-inflammatory activities—reduces NSCLC cell proliferation and migration, potentially via ERRα pathway modulation [20]. Notably, recent studies on edible fungi have also reported prominent anticancer effects against A549 cells: for instance, Agrocybe praecox exhibits significant inhibitory activity on A549 cell viability, which may be attributed to its high total phenol content and strong antioxidant capacity that synergistically regulate tumor cell redox balance [21]. This further supports that natural products with antioxidant and phenol-rich properties could be potential adjuvants in NSCLC treatment, complementing the therapeutic effects of our identified core herbal components.

Through PPI network analysis, TP53, AKT1, and EGFR were identified as core target proteins in NSCLC pathogenesis. TP53, a critical tumor suppressor gene frequently mutated in NSCLC, is dysregulated, leading to impaired apoptosis and enhanced tumor angiogenesis and proliferation [22]. AKT1, a key node in the PI3K/AKT pathway, is hyperactivated in tumors to promote cell survival and proliferation [23]. Studies show AKT1 reduces NSCLC chemosensitivity and facilitates invasion/migration in EGFR/K-RAS-mutated cells [24]. EGFR overexpression/mutation is a defining feature of NSCLC, underpinning EGFR-targeted therapy [25]. The *Astragali Radix*-*Poria* combination likely inhibits NSCLC via multi-pathway modulation of these core targets, either directly or indirectly.

The findings of this study suggest that the *Astragali Radix*-*Poria* combination modulates multiple biological processes in NSCLC, including inflammatory response, hypoxic adaptation, and apoptosis regulation, thereby improving the tumor microenvironment and inhibiting tumor cell growth and metastasis. These processes are intricately linked to immune cell infiltration, implying that the herbal combination may enhance antitumor immunity by influencing immune cell recruitment and functional polarization.

GO and KEGG pathway analyses identified signaling networks critical for tumor immune surveillance and evasion, with the TNF signaling pathway exhibiting a prominent role. This pathway is known to regulate immune cell infiltration by shaping the tumor microenvironment and modulating immune cell activation. Consequently, therapeutic strategies targeting these signaling pathways may enhance immune cell infiltration into the tumor, potentially improving patient response to immunotherapy.

In this study, several genes significantly differentially expressed in LUAD were identified, including CCNA2, CDK1, and EGFR. Elevated expression of CCNA2 and CDK1—key molecules in cell cycle regulation—is strongly associated with tumor cell proliferation [26,27]. Overactivation of EGFR has been linked to enhanced tumor cell survival, proliferation, and migration [28], making EGFR-targeted therapies a valuable approach in NSCLC treatment [29]. Alterations in FOS expression have been associated with malignant phenotypic changes in tumor cells [30], while downregulation of CCL2 and CAV1 may influence immune cell recruitment and signaling within the tumor microenvironment, thereby affecting tumor immune evasion and progression [31,32]. These targets not only play critical roles in tumorigenesis but may also modulate the tumor microenvironment by influencing T-cell infiltration.

Our study revealed a significant increase in activated memory CD4^+^ T cells and plasma cells within the tumor microenvironment of NSCLC patients. Conversely, a relative decrease was observed in resting memory CD4^+^ T cells, resting NK cells, and monocytes, highlighting the distinct immune landscape of the NSCLC tumor microenvironment. Furthermore, core gene expression exhibited positive correlations with T cell infiltration, suggesting their potential involvement in NSCLC immunoregulation by promoting T cell recruitment and function.

Further analysis of the correlation between core gene expression and immune cell infiltration demonstrated significant positive associations between several key genes and diverse immune cell subsets. These findings not only underscore the intricate interplay between gene expression and immune infiltration but also provide novel insights for NSCLC immunotherapy. For example, EGFR expression was positively correlated with infiltration of CD4^+^ T cells and B lymphocytes, indicating a critical role for EGFR in NSCLC immune modulation. Thus, targeting EGFR and its downstream signaling pathways may enhance T cell infiltration and improve patient response to immunotherapy.

In summary, this study systematically evaluated the clinical efficacy of Yiqi and blood-activating therapy combined with chemotherapy for NSCLC using bioinformatics techniques including meta-analysis and data mining. The results showed that this combined approach significantly improves the short-term response rate in NSCLC patients, alleviates clinical symptoms, reduces adverse reaction incidence, and enhances overall quality of life. Additionally, the therapy exhibited notable immunomodulatory effects closely associated with core genes such as TP53, AKT1, and JUN. Mechanistically, Yiqi and blood-activating therapy significantly regulated the balance of CD4^+^/CD8^+^ T cells, thereby enhancing antitumor immune response. These findings provide new therapeutic perspectives for patients and a robust foundation for future research.

However, the study has limitations, such as small sample sizes in some included studies and incomplete descriptions of study designs, necessitating further validation through high-quality, large-sample RCTs. Notably, three additional limitations warrant acknowledgment. Most included studies were retrieved from Chinese-language databases (e.g., CNKI, Wanfang), which may introduce publication bias given the underrepresentation of eligible English-language research, potentially skewing the synthesis of efficacy data. The meta-analysis also lacked key long-term survival endpoints, specifically overall survival (OS) and progression-free survival (PFS), precluding a comprehensive assessment of the therapy’s long-term therapeutic benefits and limiting its clinical relevance for long-term NSCLC management. Furthermore, despite efforts to standardize herbal nomenclature, inconsistencies in the quality (e.g., active component content) and dosage of herbal preparations across included studies may have introduced unmeasured confounding, as such variations can directly impact therapeutic efficacy and adverse reaction profiles. Moreover, significant clinical heterogeneity was observed across studies, including variations in the composition, administration, and dosage of Chinese herbal medicines in the formulas. Future research should focus on specific tumor stages, disease courses, and treatment protocols to establish a more reliable understanding of the mechanisms of TCM in NSCLC treatment.

To simplify the standardization of herbal formulas for large-scale clinical application, our findings provide actionable insights: prioritize the core combination (*Astragali Radix*, *Atractylodis Macrocephalae Rhizoma*, *Poria cocos*) identified via data mining to reduce formula complexity while preserving efficacy, and establish unified quality criteria for key active components (e.g., quercetin, kaempferol) validated by network pharmacology to ensure batch consistency. Future multi-center trials and digital quality tools (e.g., herbal fingerprinting) for these simplified formulas will further accelerate clinical translation.

## 4. Information and Methods

### 4.1. Meta-Analysis and Data Mining

#### 4.1.1. Literature Search

This study identified published RCTs on NSCLC treated with the method of Yiqi and blood-activating therapy in combination with chemotherapy. We searched five databases for relevant articles: Web of Science, PubMed, China National Knowledge Infrastructure (CNKI), Wipro Chinese Science and Technology Journal Full Text Database (VIP), and Wanfang Database. The search period ranged from the inception of each database to January 2025. The primary search terms included non-small cell lung cancer, traditional Chinese medicine, Yiqi and blood-activating therapy chemotherapy, clinical research, and randomized controlled trial. A combination of subject and free-word searches was employed, and duplicates were eliminated.

#### 4.1.2. Study Selection

Two authors (Lu Xu and Weiling Lv) independently reviewed all retrieved articles. Initially, irrelevant studies was excluded based on titles and abstracts. Subsequently, the final articles were identified according to the inclusion and exclusion criteria. Disagreements were resolved through consultation with the corresponding author (Fan Lin) and thorough discussion. The specific criteria were as follows:

Inclusion Criteria were:

(1) Clinical RCTs with a clear and feasible randomization method.
(2) Patients with clinical manifestations of chest distension and pain, cough, hemoptysis, low-grade fever, weight loss, and respiratory distress, confirmed as NSCLC by pathological and histological testing.
(3) The experimental group received Yiqi and blood-activating Chinese herbal medicine (with a clear drug combination and dosage instructions) combined with chemotherapy, with a treatment course of at least 14 days.
(4) The control group received a clinically approved conventional chemotherapy regimen, with or without a placebo.

Exclusion Criteria were:

(1) Non-RCT studies.
(2) Studies that did not meet the diagnostic criteria for primary NSCLC.
(3) Studies without definitive pathological or cytological diagnosis.
(4) Interventions that were not in line with the study protocol, such as treatments other than non-oral Chinese medicine (e.g., acupuncture, moxibustion, cupping) or anti-tumor treatments other than chemotherapy (e.g., targeted therapy).
(5) Patients with NSCLC accompanied by other serious primary diseases or complications.
(6) Studies with incomplete or unclear outcome data, or those with duplicate publications.
(7) Sample sizes smaller than 30.

#### 4.1.3. Efficacy and Outcome Measures

(1) Recent Efficacy: Objective response rate (ORR) = (CR + PR)/total cases × 100%, where complete response (CR) is defined as the complete disappearance of lesions on imaging, maintained for at least 4 weeks, and partial response (PR) is defined as lesion shrinkage > 50% with no new lesions appearing.
(2) Clinical Symptom Efficacy: Based on the Guiding Principles for Clinical Research of New Chinese Medicines, symptoms such as cough and phlegm, chest pain and hemoptysis, fatigue, emaciation, loss of appetite, and dark complexion were scored on a scale of 0 to 3 (none, mild, moderate, severe). The TCM symptom score ratio was calculated as follows: TCM symptom score ratio = [(total TCM symptom score before treatment − total TCM symptom score after treatment)/total symptom score before treatment] × 100%. Clinical efficacy was categorized as follows: significant effect (70% ≤ TCM symptom score ratio < 100%), effective (30% ≤ TCM symptom score ratio < 70%), and ineffective (<30%).
(3) Incidence of Adverse Reactions: White blood cell decline, platelet decline, and incidence of gastrointestinal reactions.
(4) Quality of Life: The KPS score was used. An increase of more than 10 points from the pre-treatment score was considered an improvement, a decrease of more than 10 points was considered a decline, and a change of no more than 10 points was considered stable.
(5) Changes in T-cell Subsets: CD3+, CD4+, CD4+/CD8+, and CD8+.

#### 4.1.4. Data Extraction, Quality Evaluation, and Meta-Analysis

Two investigators (LuXu and Weiling Lv) independently extracted detailed information from 57 articles, including the following: (1) basic information of the included studies (first author’s name, year of publication); (2) characteristics of subjects at baseline and after treatment (sample size, treatment regimen, composition of herbal medicines, and duration of treatment); and (3) detailed endpoints (recent efficacy, clinical symptom efficacy, incidence rate of adverse reactions, quality of life, and changes in T-cell subsets).

The quality of the studies was assessed using the Cochrane Risk of Bias (ROB) tool (version 5.1), with studies rated as ‘High,’ ‘Unclear,’ or ‘Low’ risk of bias. In cases of disagreement, a third party was consulted for judgment. The following items were evaluated: (1) random sequence generation, (2) allocation concealment, (3) blinding of participants and personnel, (4) blinding of outcome assessment, (5) incomplete outcome data, (6) selective reporting, and (7) other biases. Studies with complete information were rated as ‘low risk,’ those with unmentioned information as ‘unclear risk,’ and those with significant bias as ‘high risk.’

Meta-analyses were performed using RevMan 5.4 software. The effect sizes for dichotomous and continuous variables was assessed using Odds Ratio (OR) and standardized mean difference (SMD), respectively. A fixed-effects model was used when heterogeneity was low (I^2^ < 50%); otherwise, a random-effects model was employed. Subgroup analyses were conducted based on different outcome measures, and potential effect modifications were assessed through sensitivity analysis. The significance level was set at 0.05, with *p* < 0.05 indicating statistical significance.

#### 4.1.5. Data Mining of Chinese Medicines

According to the standardization guidelines of the Chinese Pharmacopoeia, the names of Chinese medicinal herbs collected from prescriptions were normalized; for example, ‘Yuanhu’ was uniformly standardized as ‘*Corydalis yanhusuo*,’ and ‘Shashen’ was standardized as ‘*Anemarrhena asphodeloides*’ (commonly known as Zhimu). Different parts of *Trichosanthes kirilowii* were retained: fruits (Gualou) and roots (Tianhuafen). Two group members independently performed these operations and cross-checked the results. The attributes of Chinese medicines were analyzed using the software ‘Ancient and Modern Medical Case Cloud Platform’ (V2.2.1). SPSS Statistics 23.0 was used for frequency analysis, and high-frequency herbs (>20%) were analyzed by systematic clustering. Connected drugs were identified based on a relative distance criterion of 20. Using SPSS Modeler 18.0, Apriori correlation analysis was conducted with a minimum support level of 15% and a minimum confidence level of 80%. The correlation diagram was plotted, and the results were exported.

### 4.2. Network Pharmacology and Immune Infiltration Analysis

#### 4.2.1. Network Pharmacological Analysis

We mined the GeneCards, OMIM, and DRUGBANK databases to identify potential targets in NSCLC. The Traditional Chinese Medicine Systems Pharmacology (TCMSP) Database and Analysis Platform was used in combination with a literature search to screen core TCM ingredients and capture their targets of action. The screening criteria were set at oral bioavailability (OB) ≥ 30% and drug-likeness (DL) ≥ 0.18. The identified targets were converted to gene names using the UniProt library. The intersection of active TCM ingredients and disease-related targets was visualized using Cytoscape software (version 3.10.2) to construct a “TCM-compound-target-disease” network diagram. The intersection targets of diseases and drugs were submitted to the STRING 11.0 database to construct a protein–protein interaction (PPI) network model. The species was set to Homo sapiens, and the minimum interaction threshold was set to “highest confidence” (>0.9). Other settings were maintained at default values to obtain the PPI network. Core targets were subjected to GO functional enrichment analysis and KEGG pathway enrichment analysis using the DAVID online database. A *p*-value of < 0.05 was considered statistically significant.

#### 4.2.2. Differentially Expressed Gene Analysis

Transcriptome sequencing data and corresponding clinical data for LUAD were downloaded from The Cancer Genome Atlas (TCGA) website. Specifically, we obtained the LUAD RNA-Seq data and the associated clinical information from the TCGA database. Differentially expressed genes (DEGs) with |log_2_FC| > 1.0 and *p* < 0.05 were identified using the R (version 4.3.3) ‘limma’ package. Heatmaps were generated using the R ‘pheatmap’ package. For significantly differentially expressed DEGs, the threshold was set at |log_2_FC| > 1.5. These DEGs were intersected with the PPI protein network, which was ranked from highest to lowest based on gene degree value.

#### 4.2.3. Differential and Correlation Analysis of Immune Cell Infiltration

The CIBERSORT algorithm was used to predict the abundance of 22 infiltrating immune cell types in LUAD tumor and normal tissue samples. The Wilcoxon signed-rank sum test was employed to compare the proportions of these 22 immune cell types between normal and tumor tissues. Pearson correlation analysis was performed to evaluate the association between immune cell infiltration and immune checkpoints. Violin plots, scatter plots, and correlation heatmaps were generated using the R software (version 4.3.3) packages ‘vioplot’, ‘ggplot2’, and ‘ggpubr’. A *p*-value of <0.01 was considered statistically significant.

The correlation between the expression of key genes and tumor purity, B-lymphocytes, CD8^+^ T-lymphocytes, CD4^+^ T-lymphocytes, macrophages, neutrophils, and dendritic cells was analyzed using the TIMER database (https://timer.comp-genomics.org/, accessed on 20 January 2025).

## Figures and Tables

**Table 1:** Meta-Analysis of Efficacy Outcome Indicators.

Efficacy Outcome Indicators	Treatment Group (Combination of Yiqi Huoxue Formula and Chemotherapy)	Control Group (Yiqi Huoxue Formula Only)	Heterogeneity Test	Meta-Analysis Results
Recent Effective Rate	357	354	I^2^ = 0%	OR = 1.44, 95% CI =(1.33, 1.66)
			*p* = 0.80	Z = 9.15 (*p* < 0.0001)
Clinical Symptom Efficacy	842	849	I^2^ = 11%	OR = 4.40, 95% CI = (3.48, 5.55)
			*p* =0.31	Z = 12.43 (*p* < 0.00001)
Incidence of Adverse	/	/	/	/
Reactions				
Leukopenia	994	983	I^2^ = 0%	OR = 0.32, 95% CI = (0.25, 0.40)
			*p* = 0.53	Z = 10.25 (*p* < 0.00001)
Thrombocytopenia	596	603	I^2^ = 0%	OR = 0.34, 95% CI = (0.24, 0.47)
			*p* = 0.83	Z = 6.34 (*p* < 0.00001)
Gastrointestinal Reaction Incidence Rate	1120	1112	I^2^ = 0%	OR = 0.39, 95% CI = (0.28, 0.53)
			*p* = 0.46	Z = 5.96 (*p* < 0.00001)
KPS Score	297	296	I^2^ = 4%	OR = 5.58, 95% CI = (4.69, 6.47)
			*p* = 0.40	Z = 12.26 (*p* < 0.00001)
T-cell Subset Changes	/	/	/	/
CD3+	382	372	I^2^ = 93%	WMD = 5.32, 95% CI = (2.05, 8.60)
			*p* < 0.00001	Z = 3.19 (*p* = 0.001)
CD4+	495	511	I^2^ = 92%	WMD = 4.97, 95% CI = (3.19, 6.74)
			*p* < 0.00001	Z = 5.48 (*p* < 0.00001)
CD8+	360	367	I^2^ = 93%	WMD = −1.52, 95% CI = (−1.94, −1.10)
			*p* < 0.00001	Z = 7.13 (*p* < 0.0001)
CD4+/CD8+	463	483	I^2^ = 93%	WMD = 0.28, 95% CI = (0.12, 0.44) Z = 3.34 (*p* = 0.008)
			*p* < 0.00001	

Note: Heterogeneity was assessed using significance tests, and the *p*-value and I^2^ statistic should be interpreted together. The core logic is to first determine the presence of heterogeneity via the *p*-value, then evaluate the magnitude of heterogeneity using the I^2^ statistic. The specific method is as follows: A *p*-value threshold is set as the judgment criterion. If the *p*-value is greater than 0.1, it indicates no significant heterogeneity among studies (i.e., homogeneity exists), and a fixed-effects model should be used for the meta-analysis. If the *p*-value is less than 0.00001, it signifies the presence of statistical heterogeneity, and a random-effects model is required to analyze the meta-analysis results. Additionally, a *p*-value less than 0.05 is generally regarded as the criterion for determining that the between-group difference is statistically significant. Next, the I^2^ statistic is used to evaluate “the magnitude of heterogeneity”, with the following judgment criteria: I^2^ = 0%: No heterogeneity; 0% < I^2^ ≤ 50%: Mild heterogeneity, which is generally acceptable; 50% < I^2^ ≤ 75%: Moderate heterogeneity; I^2^ > 75%: High heterogeneity. Abbreviations: OR = Odds Ratio, CI = Confidence Interval, I^2^ = Percentage heterogeneity (I-squared statistic), *p* = *p*-value for heterogeneity test, Z = Z-value for meta-analysis effect size, WMD = Weighted Mean Difference, KPS = Karnofsky Performance Status, CD3+, CD4+, CD8+ = T cell subsets (Cluster of Differentiation markers).

**Table 2:** Commonly Used Chinese Medicinal Plant Formulations for the Clinical Treatment of NSCLC.

Items (Left-Hand Side ≥ Right-Hand Side)		Support	Confidence
*Astragali Radix* (Huangqi)	*Atractylodis Macrocephalae Rhizome* (Baizhu)	45.283	100
*Astragali Radix* (Huangqi)	*Codonopsis Radix* (Dangshen)	39.623	90.476
*Astragali Radix* (Huangqi)	*Poria* (Fuling)	35.849	100
*Astragali Radix* (Huangqi)	*Hedyotidis Diffusae Herba* (Baihuasheshecao)	35.849	94.737
*Astragali Radix* (Huangqi)	*Atractylodis Macrocephalae Rhizome* (Baizhu), *Poria*(Fuling)	32.075	100

**Table 3:** Profiling of Core Gene Expression in NSCLC).

Gene Name	Variance Multiplier	Trends in Expression	*p*-Value
CCNA2	3.936589237	upwards	2.15 × 10^−31^
CDK1	3.482888292	upwards	2.85 × 10^−31^
EGFR	1.642753191	upwards	1.18 × 10^−9^
FOS	−2.350464865	downward	1.31 × 10^−22^
CCL2	−2.374968413	downward	5.73 × 10^−15^
CAV1	−3.141735087	downward	1.01 × 10^−30^

## Data Availability

The datasets involved in our study were extracted from CNKI (http://www.cnki.net/, accessed on 15 January 2025), VIP (https://qikan.cqvip.com/, accessed on 15 January 2025), Wanfang (http://www.wanfangdata.com.cn, accessed on 15 January 2025), and PubMed (https://pubmed.ncbi.nlm.nih.gov/, accessed on 15 January 2025). For additional information, please contact the corresponding authors.

## Abbreviations

The following abbreviations are used in this manuscript:

TCM	Traditional Chinese medicine
NSCLC	non-small cell lung cancer
CNKI	China National Knowledge Infrastructure
VIP	Wipro Chinese Science and Technology Journal Full Text Database
RCTs	Randomized Controlled Trials
KPS	Karnofsky Performance Status
TCMP	Traditional Chinese Medicine Pharmaceutical
HQ	Scutellaria baicalensis
FL	Poria cocos
BZ	Atractylodes macrocephala
PPI	protein–protein interaction
GO	Gene Ontology
BP	biological processes
CC	cellular components
MF	molecular functions
KEGG	Kyoto Encyclopedia of Genes and Genomes
LUAD	Lung adenocarcinoma
TCGA	The Cancer Genome Atlas
DEGs	differentially expressed genes
NK	natural killer
ORR	Objective response rate
CR	complete remission
PR	partial remission
OR	Odds Ratio
SMD	standardized mean difference
TCMSP	Traditional Chinese Medicine Systems Pharmacology
OB	oral bioavailability
DL	drug-likeness

## Appendix A

**Table A1:** Research characteristics of included studies.

Included Studies	Sample Size		Interventions		Treatment Duration	Outcome Measure
	Experimental Group	Control Group	Experimental Group	Control Group		
Zhang, 2020 [33]	30	28	Fuzheng and blood stasis prescription + chemotherapy	GP	42 d	1, 2, 3, 5
Sun, 2019 [34]	29	28	Fuzheng tumor suppression soup + DP	DP	84 d	2, 3, 4
Lu, 2016 [35]	18	22	Tumor by stasis + chemotherapy	AP/GP	60 d	2, 3, 4
Li, 2017 [36]	33	33	Promoting blood circulation and removing blood stasis with prescription + chemotherapy	Chemotherapy with cisplatin alone	28 d	1, 5
Zhou, 2015 [37]	20	20	Blood house by yu pills + chemotherapy	TP	42 d	1
Wang, 2022 [38]	56	56	Compound lung detoxification prescription + chemotherapy	GP/PP/TP	168 d	1, 2, 4
Wu, 2021 [39]	35	35	Peiyuan anticancer soup + TP	TP	84 d	1, 5
Zhang, 2021 [40]	33	35	Qilian mixture + chemotherapy	AP/TP/GP	84 d	1, 2, 3
Gao, 2022 [41]	63	63	Raw vein drink blood house by blood stasis soup + chemotherapy	Conventional chemotherapy	42 d	1, 3
Wang, 2015 [42]	27	27	Warm Yang qi and blood circulation prescription + chemotherapy	NP/GP	42 d	1, 2, 3, 4
Cheng, 2016 [43]	30	30	Blood house by stasis particles add flavor + chemotherapy	DC	21 d	2, 3
Li, 2020 [44]	30	30	Blood house by blood stasis soup + chemotherapy	GP/PC	42 d	2, 4
Li, 2018 [45]	35	34	Blood house by blood stasis soup + chemotherapy	GP	63 d	1
Wang, 2019 [46]	40	40	Yiqi solid elimination of cancer prescription + chemotherapy	NP/PT/PD/PG	28 d	4, 5
He, 2017 [47]	40	40	Benefit elimination formula + chemotherapy	GP	84 d	4
Li, 2017 [48]	51	51	Yiqi and blood stasis prescription + chemotherapy	TP	63 d	1, 4
Shen, 2017 [49]	40	40	Yiqi and blood stasis prescription + chemotherapy	DP	60 d	2
Ren, 2019 [50]	85	85	Yiqi and blood stasis prescription + chemotherapy	TP	84 d	1, 3
Yang, 2021 [51]	51	51	Yiqi Hua yu soup he sand ginseng ophiopogon soup + chemotherapy	GP	84 d	1, 4, 5
Wang, 2023 [52]	26	26	Yiqi Hua yu soup he sand ginseng ophiopogon soup + chemotherapy	GP	32 d	1, 5
Wei, 2015 [53]	30	30	Qi and blood circulation prescription + chemotherapy	GP	63 d	3, 4
Wu, 2017 [54]	30	30	Qi and blood circulation prescription + chemotherapy		56 d	1, 2, 3, 4
Chen, 2019 [55]	30	30	Benefit qi, promote blood circulation and relieve prescription + chemotherapy	PC	21 d	2, 5
Li, 2018 [56]	48	48	Qi blood soup + chemotherapy	NP/GP/TP/PC	28 d	1
Sun, 2020 [57]	30	30	Qi and blood circulation and tumor elimination soup + chemotherapy	DP	84 d	4, 5
Liu, 2022 [58]	48	48	Yiqi, anticancer and blood circulation prescription + chemotherapy	TP	120 d	2, 4
Huang, 2018 [59]	45	45	Benefiting qi, nourishing Yin and removing blood stasis method + chemotherapy	GP	63 d	2
Tang, 2020 [60]	35	35	Distasis prescription + chemotherapy	PC/GP/NP	14 d	1, 2, 3, 4
Zhu, 2017 [61]	31	31	Qi, clearing, poison and removing blood stasis + chemotherapy	TP	42 d	1
Zhou, 2013 [62]	45	48	Qi and blood circulation prescription + chemotherapy	NP	63 d	1, 4
Xiong, 2012 [63]	39	39	Qi, clearing, poison and removing blood stasis + chemotherapy	NP	42 d	1, 2, 4
Han, 2012 [64]	35	25	Fuizer prescription + chemotherapy	TP	90 d	1
Ding, 2012 [65]	43	43	Benefit qi and nourish blood, eliminate the accumulation of traditional Chinese medicine + chemotherapy	NP	56 d	1
Chen, 2012 [66]	38	38	Qi blood soup + chemotherapy	TP	42 d	1
Song, 2011 [67]	32	34	Qi elimination soup + chemotherapy	PD	20 d	1, 4
Qi, 2011 [68]	30	30	The rhubarb concubine worm pill + chemotherapy	TP	42 d	1, 3, 4, 5
Tang, 2010 [69]	38	38	Benefiting qi, nourishing lung, removing phlegm, removing blood stasis and detoxification, total + chemotherapy	GP	180 d	1, 2
Zhang, 2010 [70]	25	20	Twelve wei tumor suppression capsule + chemotherapy	GP/NP	42 d	1, 4
Chen, 2010 [71]	30	28	Fuzhengoma soup + chemotherapy	TP	42 d	1, 4
Liu, 2010 [72]	50	48	Qi and blood circulation prescription + chemotherapy	NP	42 d	1, 2, 4
Zhang, 2010 [73]	38	36	Compound Wubone vine soup + chemotherapy	NP	42 d	1, 4, 5
Liu, 2009 [74]	30	26	Twelve wei tumor suppression capsule + chemotherapy	GP	42 d	1, 4
Xiang, 2008 [75]	52	40	Qi blood soup + chemotherapy	EP	64 d	1
Zheng, 2007 [76]	38	40	Lung tumor flat cream + chemotherapy	NP/EP	42 d	1, 2
Deng, 2023 [77]	44	43	Supplementing Lung and Dispelling Accumulation Decoction + chemotherapy	BVZ	21 d	1, 3, 4
Xi, 2023 [78]	35	34	Astragalus and Ginseng Soup for Dispelling Masses + chemotherapy	NP/NDP/CAM	28 d	3, 4, 5
Xu, 2024 [79]	226	222	Fuzheng-Quxie Formula + chemotherapy	EGFR-TKI	28 d	3
Cho, 2025 [80]	35	35	Buzhong Yiqi Tang + chemotherapy	PEM	315 d	3, 4

Note: Outcome Measures Include: 1. Recent Efficacy, 2. Clinical Symptomatic Efficacy, 3. Incidence of Adverse Effects, 4. Karnofsky Performance Status (KPS) Score, 5. Changes in T-Cell Subsets.

## References

[1] George J.E., George P.S., Krishna J.K.M., Mathew A. (2025). Global trends in lung cancer incidence and mortality by age, gender and morphology and forecast: A bootstrap-based analysis. Lung Cancer.

[2] Zheng R.S., Zhang S.W., Sun K.X., Chen R., Wang S.M., Li L., Zeng H.M., Wei W.W., He J. (2023). Cancer statistics in China, 2016. Zhonghua Zhong Liu Za Zhi.

[3] Czerwińska M., Kuśnierek M. (2024). Lycium barbarum fruits—Phytochemistry and activity of goji berries—From tradition to clinical studies. Prospect. Pharm. Sci..

[4] Liu Y., Fang C., Luo J., Gong C., Wang L., Zhu S. (2024). Traditional Chinese Medicine for Cancer Treatment. Am. J. Chin. Med..

[5] Cumpston M., Li T., Page M.J., Chandler J., Welch V.A., Higgins J.P., Thomas J. (2019). Updated guidance for trusted systematic reviews: A new edition of the Cochrane Handbook for Systematic Reviews of Interventions. Cochrane Database Syst. Rev..

[6] Tran M.N., Kim S., Nguyen Q.H.N., Lee S. (2022). Molecular Mechanisms Underlying Qi-Invigorating Effects in Traditional Medicine: Network Pharmacology-Based Study on the Unique Functions of Qi-Invigorating Herb Group. Plants.

[7] Jia D., Wang W.P. (2019). Analysis of distribution law of TCM syndrome types of patients with primary lung cancer. Shanxi J. Tradit. Chin. Med..

[8] Guo M. (2018). Research Progress on Qi Deficiency and Blood Stasis Syndrome in Patients with Advanced Non-Small Cell Lung Cancer. Chin. Mod. Med..

[9] Cui H.Z., Zhu L., Niu Q., Guo J.Z. (2010). Clinical observation of Shenqi Fuzheng Injection combined with chemotherapy in the treatment of non-small cell lung cancer. J. Mod. Oncol..

[10] Wang S.S. (2016). Impact of Gong’an Guzhen Decoction on Hemorheology in Lung Cancer Patients with Qi Deficiency and Blood Stasis Syndrome. Asia-Pac. Tradit. Med..

[11] Lu T.C., Li J., Zhu G.H., Wu J.Y., Yu J.W., Zhu X.Y., Xu B.W., Wang H.P. (2021). Study on difference of effect of Huoxue drugs and Yiqi Huoxue drugs on tumor metastasis based on immune remodeling. China J. Chin. Mater. Medica.

[12] Li G.X., Zheng Q.M., Li Y.Y., Wang Y.P., Zhang M., Sun Z.G. (2025). Correlation of peripheral blood CD4/CD8 ratio with efficacy and prognosis in patients with non-small cell lung cancer receiving PD-1/PD-L1 inhibitors. J. Coll. Physicians Surg. Pak..

[13] Song D.S., Pang Z.Y. (2024). Study on the Treatment of Advanced Non-Small Cell Lung Cancer with Qi-Deficiency and Phlegm-Dampness Syndrome Using Yiqi Huatan Formula Combined with Gefitinib Tablets. Tradit. Chin. Med. Res..

[14] Tang X.L.Z., Fang C., Meng J., Li J., Zhuang J.N. (2019). Experience of National Medical Master Zhou Daihan in Treating Lung Cancer with Xingxia Ditan Decoction. Guid. J. Tradit. Chin. Med. Pharmacol..

[15] Peng C., Chen J., Cui W., Li S., Li J., Peng L. (2022). Comparative efficacy of various CHIs combined with western medicine for non-small cell lung cancer: A bayesian network meta-analysis of randomized controlled trials. Front. Pharmacol..

[16] Su K., Xu P., Bai Q. (2024). Shenling Baizhu Powder Combined with EGFR-TKIs Targeted Treatment for Non-small Cell Lung Cancer. Guangming J. Chin. Med..

[17] Yang W.G., Zhang P.W., Chen L., Deng Y., Shi J., Yan S. (2020). Clinical Discussion on Oral Administration of Modified Guizhi Fuling Pills for Patients with Lung Cancer. World J. Complex Med..

[18] Alduais Y., Zhang H., Fan F., Chen J., Chen B. (2023). Non-small cell lung cancer (NSCLC): A review of risk factors, diagnosis, and treatment. Medicine.

[19] Li H.Y., Xu J., Na H., Wang S.H. (2017). Effects of quercetin on suppressing migration and invasion of A549 cells via the STAT3 signaling pathway. J. Int. Pharm. Res..

[20] Zhang J., Shi X., Meng W., Ma F., Zhao L., Zhao J. (2018). Kaempferol inhibits invasion and migration of non-small cell lung cancer A549 cells by down-regulating ERRα expression. Chin. J. Cancer Biother..

[21] Korkmaz A.İ., Bal C., Eraslan E.C., Sevindik M., Akgul H. (2023). Biological activities of Agrocybe praecox (spring fieldcap mushroom). Prospect. Pharm. Sci..

[22] Lin W., Yan Y., Huang Q., Zheng D. (2024). MDMX in Cancer: A Partner of p53 and a p53-Independent Effector. Biol. Targets Ther..

[23] de Langen A.J., Johnson M.L., Mazieres J., Dingemans A.C., Mountzios G., Pless M., Wolf J., Schuler M., Lena H., Skoulidis F. (2023). Sotorasib versus docetaxel for previously treated non-small-cell lung cancer with KRAS(G12C) mutation: A randomised, open-label, phase 3 trial. Lancet.

[24] Wu J., Lin Z. (2022). Non-Small Cell Lung Cancer Targeted Therapy: Drugs and Mechanisms of Drug Resistance. Int. J. Mol. Sci..

[25] Huang H.Y., Xu Z.W.D., Xia L.L., Yu Y.F., Lu S. (2023). Research Progress on Immunotherapy for Advanced Non-Small Cell Lung Cancer with Epidermal Growth Factor Receptor Mutation. J. Shanghai Jiaotong Univ. (Med. Sci.).

[26] Luo J., Mo X., Hu D., Li Y., Xu M. (2024). New perspectives on the potential of tetrandrine in the treatment of non-small cell lung cancer: Bioinformatics, Mendelian randomization study and experimental investigation. Aging.

[27] Xie M.Y., Ding K., Jing X.M., Wei Y.T., Shu Y.Q. (2022). Identification and verification of hub genes associated with the progression of non-small cell lung cancer by integrated analysis. Front. Pharmacol..

[28] Meng Y., Bai R., Cui J. (2023). Precision targeted therapy for EGFR mutation-positive NSCLC: Dilemmas and coping strategies. Thorac. Cancer.

[29] Fu K., Xie F., Wang F., Fu L. (2022). Therapeutic strategies for EGFR-mutated non-small cell lung cancer patients with osimertinib resistance. J. Hematol. Oncol..

[30] Chen Y., Song W., Gao Y., Dong X., Ji X. (2022). Increased PD-L1 Expression in Acquired Cisplatin-Resistant Lung Cancer Cells via Mir-181a. Tohoku J. Exp. Med..

[31] Xu H., Wang J., Al-Nusaif M., Ma H., Le W. (2024). CCL2 promotes metastasis and epithelial-mesenchymal transition of non-small cell lung cancer via PI3K/Akt/mTOR and autophagy pathways. Cell Prolif..

[32] Cui S., Lou S., Feng J., Tang X., Xiao X., Huang R., Guo W., Zhou Y., Huang F. (2022). Identification of genes and pathways leading to poor prognosis of non-small cell lung cancer using integrated bioinformatics analysis. Transl. Cancer Res..

[33] Zhang Y., Yang N., Cao D., Bai L., Chang S., Ren G. (2020). Clinical study of Fuzheng Huayu prescription combined with chemotherapy in the treatment of qi-deficiency and blood-stasis syndrome in advanced non-small cell lung cancer. Hebei J. Tradit. Chin. Med..

[34] Sun A. (2019). Clinical Observation of Fuzheng Yiliu Decoction in the Treatment of Qi-Deficiency and Blood-Stasis Non-Small Cell Lung Cancer Patients Undergoing Chemotherapy. Master’s Thesis.

[35] Lu M. (2016). Theoretical and Applied Study of Hualiu Zhuyu Powder Combined with Chemotherapy in Advanced NSCLC. Master’s Thesis.

[36] Li J., Li T. (2017). Clinical study of Huoxue Huayu decoction combined with cisplatin in advanced non-small cell lung cancer. Acta Chin. Med..

[37] Zhou C. (2015). Clinical efficacy analysis of blood-activating and stasis-removing method combined with chemotherapy in advanced non-small cell lung cancer. Chin. Rural Health.

[38] Wang Y. (2022). Clinical and Metabolomics Study of Fufei Jiedu Decoction in the Treatment of Non-Small Cell Lung Cancer Based on “Cancer-Toxin” Pathogenesis Theory. Master’s Thesis.

[39] Wu Z., Li T., Si H. (2021). Effect of Peiyuan Kang’ai decoction combined with TP regimen on immune function, MMP-9, CYFRA21-1 and CAFs-positive rate in qi-deficiency and blood-stasis advanced lung cancer. Clin. Med. Res. Pract..

[40] Zhang Z. (2021). Clinical Efficacy of Qilian Mixture in Qi-Deficiency and Blood-Stasis Advanced NSCLC and Screening of Active Components Inhibiting Cell Migration. Master’s Thesis.

[41] Gao H., Wei N. (2022). Effect of Shengmai Yin combined with Xuefu Zhuyu decoction on hypercoagulable state in NSCLC patients. Shenzhen J. Integr. Tradit. Chin. West. Med..

[42] Wang X. (2015). Clinical Observation of Warming-Yang, Replenishing-Qi and Activating-Blood Method Combined with Chemotherapy in Advanced Non-Small Cell Lung Cancer. Master’s Thesis.

[43] Cheng W., Zeng Q., Wei C., Huang H., Liang J., Mo Z. (2016). Effect of modified Xuefu Zhuyu granules on coagulation mechanism in lung cancer patients with qi-deficiency and blood-stasis syndrome. J. Liaoning Univ. Tradit. Chin. Med..

[44] Li J. (2020). Clinical Study of Xuefu Zhuyu Decoction in Improving Hypercoagulable State in Advanced Non-Small Cell Lung Cancer Patients with Blood-Stasis Obstructing Lung Syndrome. Master’s Thesis.

[45] Li Y., Liu H.M., Jiang L.F., Liu X.L., Pei J.W., Fu B.F. (2018). Effect of modified Xuefu Zhuyu decoction on quality of life in hypercoagulable qi-deficiency and blood-stasis non-small cell lung cancer patients. J. Community Med..

[46] Wang D., Li J., Yu Z., Wang L., Zhang J., Chen S. (2019). Randomized parallel-controlled study of Yiqi Guben Xiao’ai decoction combined with chemotherapy in elderly non-small cell lung cancer (qi-deficiency and blood-stasis). J. Pract. Tradit. Chin. Intern. Med..

[47] He D., Jiang L. (2017). Effect of Yifei Xiaoji decoction on serum tumor markers and quality of life in advanced non-small cell lung cancer patients. Acta Chin. Med..

[48] Li H. (2017). Effect of yiqi huayu method on quality of life in lung cancer patients. Guangming J. Chin. Med..

[49] Shen H., Zhou Y. (2017). Clinical observation of yiqi huayu method in advanced non-small cell lung cancer. Asia-Pac. Tradit. Med..

[50] Ren B., Li Y. (2019). Clinical study of yiqi huayu decoction combined with chemotherapy in advanced non-small cell lung cancer. Shaanxi J. Tradit. Chin. Med..

[51] Yang X., Lü H., Wang H., Liang L., Wang D., Zheng L. (2021). Effect of yiqi huayu decoction plus Shashen Maidong decoction assisted chemotherapy on tumor control rate and physical status in qi-yin deficiency lung cancer patients. Pract. Pharm. Clin. Rem..

[52] Wang S. (2023). Observation on efficacy of yiqi huayu decoction combined with Shashen Maidong decoction during chemotherapy in lung cancer patients with qi-yin deficiency. Chin. J. Naturop..

[53] Wei S. (2015). Clinical Study of Yiqi Huoxue Method in Improving Quality of Life in Elderly Advanced Non-Small Cell Lung Cancer Patients Undergoing Chemotherapy. Master’s Thesis.

[54] Wu Z., Peng H. (2017). Treatment of 15 non-small cell lung cancer cases with Leng’e Shenqi decoction. Sichuan J. Tradit. Chin. Med..

[55] Chen F., Li K., Xu S. (2019). Effect of yiqi huoxue jieyu method on anxiety, depression and cellular immune function in advanced lung cancer patients. Mod. J. Integr. Tradit. Chin. West. Med..

[56] Li C. (2018). Clinical study of yiqi huoxue decoction in advanced non-small cell lung cancer. Spec. Health.

[57] Sun Y., Zou X., Zhang W. (2020). Clinical efficacy of yiqi huoxue xiaoliu decoction combined with DP regimen in advanced lung cancer. Inner Mongolia J. Tradit. Chin. Med..

[58] Liu F., Wang K., Zhou W. (2022). Efficacy observation of yiqi kang’ai huoxue decoction combined with concurrent chemoradiotherapy in qi-deficiency and blood-stasis non-small cell lung cancer. World J. Integr. Tradit. West. Med..

[59] Huang Q. (2018). Clinical study of yiqi yangyin huayu method in non-small cell lung cancer patients. Asia-Pac. Tradit. Med..

[60] Tang Z. (2020). Clinical Observation of Huayu Tongluo Decoction on Hypercoagulable State and Short-Term Chemotherapy Efficacy in Advanced NSCLC Patients. Master’s Thesis.

[61] Zhu Y. (2017). Clinical observation of TCM yiqi qingdu huayu method in advanced non-small cell lung cancer. Med. Front..

[62] Zhou Y. (2013). Clinical study of TCM yiqi huoxue decoction combined with vinorelbine and cisplatin in advanced non-small cell lung cancer. Chin. J. Clin. Oncol. Rehabil..

[63] Xiong M., Tang X., Yu J., Xiong L., Zhu H., Zeng R. (2012). Clinical observation of TCM yiqi qingdu huayu method in advanced non-small cell lung cancer. Chin. J. Tradit. Chin. Med. Pharm..

[64] Han C. (2012). Controlled observation of Fuzheng Xiaoli decoction combined with chemotherapy in advanced non-small cell lung cancer. J. Pract. Tradit. Chin. Intern. Med..

[65] Ding R. (2012). Clinical observation of integrated traditional Chinese and Western medicine in advanced lung cancer. Strait Pharm. J..

[66] Chen H., Wang W. (2012). Role of yiqi huoxue Chinese medicine in advanced non-small cell lung cancer treatment. Chin. J. Gerontol..

[67] Song C., Zhang Y., Feng Y., Zhu L. (2011). Yiqi huoxue method combined with chemotherapy in 66 advanced non-small cell lung cancer cases. Henan Tradit. Chin. Med..

[68] Qi Y., Li X., Li H. (2011). Clinical study of Dahuang Zhechong pill in lung cancer. Chin. J. Inf. Tradit. Chin. Med..

[69] Tang X. (2010). Clinical observation of yiqi yangfei quyu huoxue jiedu method in 38 phlegm-blood stasis lung cancer cases. New Chin. Med..

[70] Zhang Y. (2010). Clinical Study of Shi’erwei Yiliu Capsule Combined with Chemotherapy on P-Glycoprotein Intervention in Advanced NSCLC. Master’s Thesis.

[71] Chen Q. (2010). Fuzheng Keliu decoction combined with TP regimen in 30 advanced non-small cell lung cancer cases. Contemp. Med..

[72] Liu S., Jia Y. (2010). Clinical observation of yiqi huoxue Chinese medicine in 50 advanced non-small cell lung cancer cases. New Chin. Med..

[73] Zhang B., Min T., Wu Q. (2010). Clinical study of compound Wuguteng decoction combined with chemotherapy in advanced non-small cell lung cancer. Clin. Med..

[74] Liu P., Zhang Y., Song J. (2009). Shi’erwei Yiliu capsule combined with GP regimen in 30 advanced non-small cell lung cancer cases. Res. Tradit. Chin. Med..

[75] Xiang L., Zhai Y., Zhang L., Xiang J., Wang H., Zhang J. (2008). Clinical observation of yiqi huoxue decoction in advanced non-small cell lung cancer. Liaoning J. Tradit. Chin. Med..

[76] Zheng H., Piao B., Hua B., Zhou D., Zhang X., Liu L. (2007). Clinical observation of Feiliuping extract in 38 advanced non-small cell lung cancer (qi-yin deficiency) patients. Chin. J. Inf. Tradit. Chin. Med..

[77] Deng K. (2023). Clinical Study of Bufei Xiaoji Decoction Combined with Bevacizumab Plus Chemotherapy in Advanced Non-Squamous Non-Small Cell Lung Cancer. Master’s Thesis.

[78] Xi W. (2023). Clinical Study of Qishen Sanjie Decoction Combined with Chemotherapy and Immunotherapy in Advanced NSCLC (Qi-Yin Deficiency). Master’s Thesis.

[79] Xu R.Z., Li Y., Zhang L., Fang Z.H., Zhao F.C., Lü X. (2024). Clinical study of Fuzheng Quxie decoction combined with EGFR-TKI in advanced lung adenocarcinoma. Oncol. Pharm..

[80] Cho E., Yi J.M., Chun J.M., Jang H., Yoon S.H., Lee S.H., Jang S.H., Park D.W., Kim S.J., Um S.W. (2025). Efficacy and safety of herbal medicine Bojungikki-tang in combination with pembrolizumab versus pembrolizumab monotherapy for stage IV non-small cell lung cancer: Study protocol for a randomized, open-label, double-arm, multicenter trial. Integr. Cancer Ther..

